# Towards automated long-term acoustic monitoring of endangered river dolphins: a case study in the Brazilian Amazon floodplains

**DOI:** 10.1038/s41598-023-36518-1

**Published:** 2023-07-27

**Authors:** Florence Erbs, Marina Gaona, Mike van der Schaar, Serge Zaugg, Emiliano Ramalho, Dorian Houser, Michel André

**Affiliations:** 1 Laboratori d’Aplicacions Bioacústiques, Universitat Politècnica de Catalunya - BarcelonaTech, Barcelona, Spain; 2 Instituto de Desenvolvimento Sustentável Mamirauá, Tefé, Brazil; 3 National Marine Mammal Foundation, San Diego, USA

**Keywords:** Conservation biology, Animal behaviour

## Abstract

Using passive acoustic monitoring (PAM) and convolutional neural networks (CNN), we monitored the movements of the two endangered Amazon River dolphin species, the boto (*Inia geoffrensis*) and the tucuxi (*Sotalia fluviatilis*) from main rivers to floodplain habitats (várzea) in the Mamirauá Reserve (Amazonas, Brazil). We detected dolphin presence in four main areas based on the classification of their echolocation clicks. Using the same method, we automatically detected boat passages to estimate a possible interaction between boat and dolphin presence. Performance of the CNN classifier was high with an average precision of 0.95 and 0.92 for echolocation clicks and boats, respectively. Peaks of acoustic activity were detected synchronously at the river entrance and channel, corresponding to dolphins seasonally entering the várzea. Additionally, the river dolphins were regularly detected inside the flooded forest, suggesting a wide dispersion of their populations inside this large area, traditionally understudied and particularly important for boto females and calves. Boats overlapped with dolphin presence 9% of the time. PAM and recent advances in classification methods bring a new insight of the river dolphins’ use of várzea habitats, which will contribute to conservation strategies of these species.

## Introduction

In recent years, the International Union for the Conservation of Nature (IUCN) reassessed the status of the two river dolphin species of the Amazon, the pink river dolphin (*Inia geoffrensis*) and the tucuxi (*Sotalia fluviatilis*) from ‘Data deficient’ to ‘Endangered’^1,2^. With these new categorizations, all five remaining river dolphin species are now officially considered threatened with extinction. This alarming situation reflects the intricate combination of direct and indirect threats posed to river dolphins worldwide where conflict with commercial fisheries (i.e. competition for resources and damages to fish nets)^3–6^ are aggravated by the high level of anthropogenic pressure on tropical freshwater ecosystems^7^. In the Amazon basin, the main direct threats on river dolphin populations are being captured for bait in the commercial fishery of piracatinga *Calophysus macropterus* and entanglements in gillnets^3,8–13^. Furthermore, disruption of hydrological connectivity through dam construction, mining, agriculture and cattle ranching is profoundly impacting river ecological functions and increasingly degrading freshwater ecosystems^14^. As a consequence, Amazon River dolphin populations are declining. Recent studies highlight an alarming population reduction of 50% every 10 years for boto and every 9 years for tucuxi^6^. Current models of population viability predict a 95% reduction of boto population within 50 years^15^. These two studies were conducted in a protected area, the Mamirauá Sustainable Development Reserve (Reserva de Desenvolvimento Sustentável Mamirauá—RDSM), where anthropogenic pressures on river dolphins are likely reduced compared to non-protected areas.

Amazonian River dolphins inhabit a unique environment characterised by radical seasonal changes in water regimes. Half of the year, large areas of the riverine forests are flooded^16^, extending the habitat of the aquatic animals from the main rivers to large areas of fringing floodplains locally called *várzea* and *igapó*. These provide access to a highly complex and resource-rich environment formed by submerged vegetation. The seasonal ‘flood pulse’ is the major factor driving the distribution and movements of many Amazonian aquatic species, including freshwater fish that undertake small-scale seasonal movements between the main rivers and floodplains for completion of their life cycle^17,18^. Fish families known to constitute the major part of the river dolphin diets, such as the Characids (Characiforms) and Doradid catfish (Siluriforms)^19^, display such synchronised lateral migrations and Amazon dolphin seasonal changes in habitat density have been related to the migration of fish^20–23^.

During the low-water season, river dolphins are concentrated in the main rivers^21,24^. Preferred habitats are confluences where two or more water streams join together (e.g. small tributaries or larger channels connect to the main rivers), bays, lakes, and river margins ^23–26^. When water levels start to rise, river dolphins follow fish movements and enter the floodplains through river channels. The botos are highly adapted to the complex and cluttered environment of the floodplains; anatomical specialisations such as unfused cervical vertebrae provide extra neck flexibility and a unique shoulder joint allows for a broader rotation range of the flippers, greatly increasing manoeuvrability^27^. The botos tend to disperse across the mosaic of newly inundated habitats, including floodplain channels, internal lakes (seasonally isolated from the rivers) and flooded forests, while the tucuxis usually remain in the deeper parts of the floodplain, i.e. occupying the river channels and small confluences^21,28–30^. Once water levels start dropping, dolphins move back to the main rivers, probably to avoid entrapment^5,21^. Botos seem to use preferentially different habitats according to their age and reproductive status. Male botos and females without calves appear to have similar habitat preferences and exhibit similar movement patterns from main rivers to the várzea^21^. On the other hand, females with calves and immatures animals spend more time in the várzea habitats (bays or small confluences, channels) than in the main river, sometimes not returning to the main rivers during the low water season, if the water level remains sufficiently high^5^.

The complexity of the floodplain habitats makes surveying the internal lakes and the flooded forest extremely challenging. Until now, the vast majority of information on river dolphin distribution has relied on boat–based visual surveys that are usually conducted along rivers. Unfortunately, inconspicuous surface behaviour and occurrence in remote areas make river dolphins difficult to monitor through visual techniques. Aerial visual monitoring methods have recently emerged as an alternative or complementary approach, with the use of unmanned aerial vehicles (drones) or non-rigid airship systems (blimp) focusing on surveying dolphins in the main rivers^31,32^. These techniques, while promising in improving count estimates^31^, are restricted to open areas and cannot be applied in the flooded forest where the tree canopy prevents visual detection from above. Satellite tracking studies can inform on movements and habitat preferences of animals^33,34^ but come with major limitations regarding cost and operational complexity, risks associated with animal capture and tag attachment^35^. A study linked mortality events with tag implants in belugas^36^, whereas Martin et al.^37]^ did not find tagging affected survival rate of the tagged botos in the RDSM. While risk levels might be partially species specific, the recently published ‘best practice guidelines for cetacean tagging’ recommends that this technique should be limited to research questions that cannot be addressed by other methods^38^.

The use of Passive Acoustic Monitoring (PAM) to conduct surveys of river dolphins takes advantage of the quasi-continuous vocal production of the river dolphins^39^, resulting in a high acoustic detectability. River dolphins produce echolocation clicks to sense their environment, to orientate and forage, as well as other vocalisations including boto-specific low frequency pulsed vocalisations^40^, and whistles^41^. Echolocation clicks are produced almost continuously and constitute a reliable, consistent, highly detectable acoustic means for investigating dolphin presence (for a review of methods and applications see^42^). PAM has been successfully applied in Amazon River dolphin studies including population distribution and habitat use^43–45^, and vocal behaviour^39,44,46–51^. With technological advances, the acoustic presence of dolphins can be detected in real time^52^. However, the Amazonian environment produces numerous challenges to signal detection and classification. The complexity of the freshwater ecosystem soundscape includes various impulsive sound sources in addition to dolphin echolocation clicks, such as cavitation noise from ship engines, rain, and high frequency stridulating insects. Additionally, the complex acoustic propagation conditions in the constrained, shallow-water environments are complicated by suspended sediment and detritus that alters signal propagation via reflection, refraction and scattering.

Several methods exist to automatically detect impulsive sounds such as echolocation clicks. Support vector machine methodology and energy-based click detectors have been used for odontocete clicks (Amazon River dolphin^43^, beaked whales^53^), some of which have been coupled with neural networks (Indo-Pacific humpback dolphins^54^)**.** Other studies have used Gaussian mixture models (GMMs) with signals represented by cepstral features^55^, entropy^56^, or Gaussian-kernel-based networks^57^ and feed forward neural networks, the first kind of artificial neural networks^58^. The most recent advances in the field of automatic classification of acoustic signals use Deep Neural Networks (DNN). For cetacean echolocation clicks, this approach has been developed for sperm whale ^59^ and other odontocete clicks^60,61^. So far, Convolutional Neural Networks (CNN), a class of DNN, have not been used for classifying river dolphin signals.

In this study we combined PAM techniques with state-of-the-art automatic classification algorithms based on CNN to monitor river dolphin presence in different floodplain habitats inside the Mamirauá Sustainable Development Reserve (RDSM) in the state of Amazonas, Brazil. Specifically, we focused on (1) developing a reliable classification model that can accurately discriminate between several types of impulsive sounds present in the floodplain soundscapes (echolocation clicks from dolphins, boat engine, and rain); (2) automatically detecting dolphin acoustic presence in large datasets from different floodplain habitats, including permanent and seasonally flooded sites; (3) identifying temporal overlap between dolphin and boat presence. The classifiers developed here will form part of the conservation strategy of the RDSM.

## Materials and methods

### Study area

**Figure 1:**
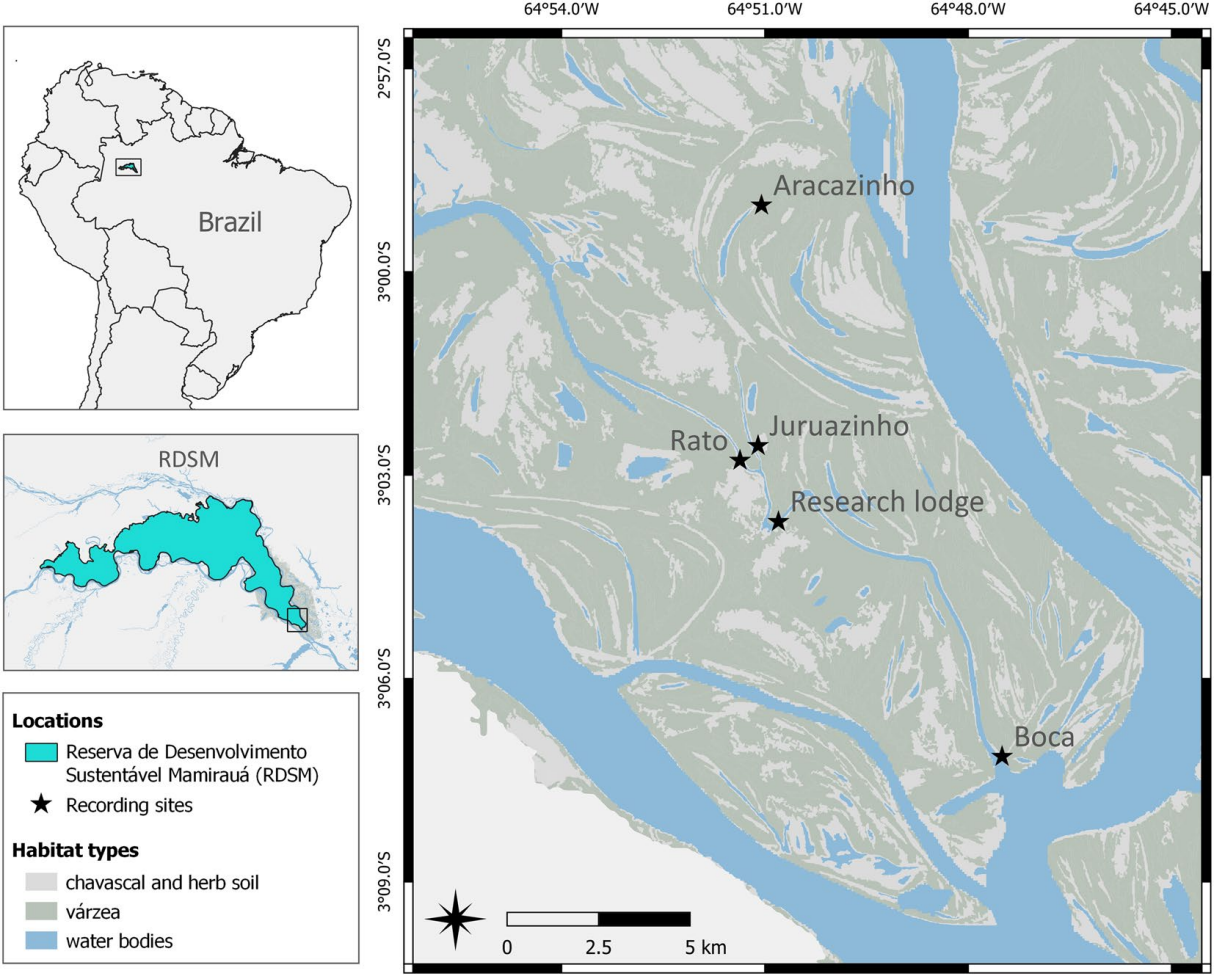
Map of the study site, the Mamirauá Sustainable Development Reserve (RDSM), Amazonas state, Brazil, and recording locations inside the RDSM. The habitat types are modified from Ferreira-Ferreira et al.^64^.

The study area covers about 800 km^2^ in the Mamirauá Sustainable Development Reserve (RDSM), in the state of Amazonas, Brazil (Fig. 1). The RSDM comprises approximately 11,000 km^2^ at the confluence of the Solimões river (upper Amazon River) and the Japurá River and is the largest Brazilian protected area dedicated to the conservation of flooded rainforests. The RDSM is inhabited by local populations along rivers and lakes, that are involved in managing and monitoring biodiversity through sustainable development. This protected status ensures that the areas under protection contain predominantly unmodified natural systems.

The region is formed by *várzea* (white-water river floodplain) habitat, a lowland forest seasonally flooded by white-waters from the Amazon, with an average annual variation in water levels of 10–12 m^62^. The region also contains patches of dense vegetation dominated by shrubs (*chavascal*) and herbaceous vegetation. During the dry season (September to March), the forest is intersected by numerous lakes and channels. During the wet season (April to August), floodwaters progressively inundate the forest, submerging most of the dry land. Two river dolphin species are present in the RDSM, the boto or pink river dolphin and the tucuxi. Each species has specific habitat preferences inside and outside the várzea (i.e. the main rivers bordered by the floodplains)^24^.

This study encompasses four different várzea habitat types comprising permanently and temporarily flooded habitats. (1) ‘*Ressacas’* are defined as shallow bays adjacent to the river channel, with low velocity current and often fringed with floating vegetation^21^. (2) River channels (*paranás*) are minor aquatic systems that connect the rivers to the floodplain lakes. (3) Internal lakes are permanently flooded systems inside the floodplain. Depending on their geomorphological type and their location, lakes display various degrees of connectivity with the main rivers and can be covered with free floating aquatic macrophytes. (4) Low várzea forests, like the flooded forest in this study, are inundated for more than 3 months of the year (as opposed to high várzea forests) and covered with trees and shrubs^63^.

### Data acquisition

The acoustic data were acquired through different recording systems. An overview of the data collection is shown in Table 1. The Providence node, funded by the National Marine Mammal Foundation (NMMF), is composed of an icListen digital hydrophone (24-bit Smart hydrophone SB2-ETH model, Ocean Sonics, Canada, sensitivity − 170 dB re 1 V/uPa) connected to a SONS-DCL real-time processing system (Sonsetc, Spain). This node was deployed from the Mamirauá floating research lodge, with direct access to the Mamirauá channel, at a depth of 5 m. The system sampled at 128 kHz with 24-bit resolution and without additional gain. Raw data from this system was transferred to Network Attached Storage (NAS) at the Uakari floating lodge (close to the research lodge) whenever the network was available. Data used from this system was recorded between July 2019 and June 2020.

**Table 1:** Overview of the data collection at the RDSM.

Recording site	Habitat type	Recorder	Latitude / longitude	Start / end date	Sampling Frequency(kHz)	Duty cycle	Recording duration (h)
Research lodge	River channel	Providence node	3°3′41.81″S	2019-06-23	128	Continuous	2121
			64°50′48.59″W	2020-06-13			
Boca	Bay	SM4	3° 7′9.60″S	2019-11-05	96	1 on/9 off	393
			64°47′30.88″W	2020-04-29			
Araçazinho	Internal lake	SM4	2°59′1.34″S	2019-07-09	192	1 on/9 off	139
			64°51′3.28″W	2019-09-05			
Juruazinho	Flooded forest	SM4	3°2′34.69″S	2019–07-09	192	1 on/9 off	139
			64°51′6.07″W	2019-09-05			
Rato	Flooded forest	SM4	3°2′47.18″S	2019-02-06	96	10 on/110 off	139
			64°51′22.13″W	2019-05-29			
Multiple boat-based surveys	River channel, bay, internal lakes	Sound Trap	Multiple sites within MSDR	2019-06-01	512	Continuous during survey	83
				2020-05-01			

Additionally, four Wildlife Acoustics SM4 recorders (Wildlife acoustics, USA) equipped with HTI-96-Min hydrophones (High Tech Inc., USA, sensitivity − 165 dB re 1 V/uPa) were deployed in different várzea habitat types. One system was located at *Boca*, a *ressaca* (or bay) at the entrance of the Mamirauá Channel (situated at 2 km from the confluence with the Japurá River), deployed from a floating house at a depth of 3–5 m, and recorded from November 2019 to April 2020. Two other systems were deployed inside the flooded forest, fixed to trees: *Rato* (between the Mamirauá channel and a depression lake) from February 2019 to May 2019; *Juruazinho* (between a *parana* and a depression lake) from July 2019 to September 2019. The last system was deployed at *Aracazinho* (a scroll lake with intermediate connectivity with the Japurá River) from July 2019 to September 2019. The latter three were deployed in areas that are not connected to the main rivers during the dry period of the year. All SM4 systems were sampled at 96 kHz with 16-bit resolution and without additional gain.

Separately from the autonomous recorders, manual recordings were collected during monthly boat-based surveys in the Mamirauá reserve, from July 2019 to May 2020. The recordings were made in close vicinity of dolphin groups (< 50 m) using a SoundTrap 300 HF (Ocean instruments, New Zealand, sensitivity − 176 dB re 1 V/uPa) deployed from the boat at 3 m depth. This data provided ground truth data for the training of the classifier as the signals came with visual identification.

### CNN classification procedure

#### Data annotation

Data labelling was performed using a Python-based custom graphical interface (labelling tool) that displayed segments of spectrograms of a given duration (here, 5 s) and allowed to annotate signal extension in time and frequency with bounding-boxes and assign sound-types (classes). Sound types were assigned only one label and multiple labels could be present independently at the segment level. The annotations were incrementally stored in a dedicated database (the Controlled Acoustic Repository database, or CAR DB). Frequency and time boundaries of each signal were then easily extracted from the bounding-boxes.

A subset (2.63 h) of data were selected from the boat-based recordings (9% manually selected to contain river dolphin signals and 91% randomly selected to include a representative sample of soundscape variability). A subset (6.57 h) of data was also randomly selected for the Providence node over a 2-day period. Both subsets of data were initially labelled with two target classes: echolocation clicks and boat engine noise, hereafter referred to as ‘*click’* and ‘*boat’* classes. Additional background sounds (e.g. aquatic insects, dolphin whistles, fish calls, …) were labelled to ensure proper representation in the training set. In addition to labelled segments, segments containing only background sounds were included. Echolocation clicks were not separated by species as there is currently no click-based method developed to discriminate between the two river dolphin species (boto and tucuxi) present in the study area. After a first training of the classifier, this initial labelling was completed by an active learning labelling (see paragraph below). An overview of the data annotation is given Table 2.

**Table 2:** Overview of annotated data used for the initial model (first 3 rows) and the final model (all 5 rows).

Dataset	Initial labelling			Active learning labelling		Total	Train Pre-DA # segments	Train DA # segments	Test #segments
	Boat-based survey	Boat-based survey	Research lodge	Research lodge	Várzea sites	All			
Selection	Manual	Automatic (random)	Automatic (10 s. per minute over 2 days)	Semi-automatic (Random above threshold)	Semi-automatic (Random above threshold)	All	NA	NA	NA
Duration of data subset (h)	0.23	2.40	6.57	0.41	1.56	11.17	NA	NA	NA
Use	Test	Train	Train/test (Equal split)	Train	Train/test (Equal split)	Train/test	NA	Train	Test
no. segm. with echolocation clicks	138	1230	96	25	76	1565	1337	4106	228
no. segm. with ship noise	0	308	104	69	178	659	530	1130	129
no. segm. with rain	0	0	0	0	378	378	188	382	190
no. segm. without any of the 3 classes (background)	30	403	4533	204	362	5532	3046	3204	2486

#### Data augmentation

Data augmentation to artificially expand the training dataset was performed in two steps, focusing on providing additional spectrograms containing soundtypes that were underrepresented in the training data. The first step was data oversampling, where labels from small classes were duplicated to have a minimum number of labels per epoch for the training. In this case, the minimum number of labels was set to 300. Duplication was done by duplicating segments that contained the underrepresented classes. Since a segment may have contained multiple labels, this potentially also duplicated labels of other classes. Second, with the duplicated data set, “on-the-fly” data augmentation was performed^65,66^. This was done by transforming the original data, including transformations in the frequency domain (small circular shift along the frequency axis), time shifts (small circular data shift along the time axis), contrast adjustments by modifying the spectrogram power, and time-warping (stretching the spectrogram along the time-axis and clipping it back to original length). For each epoch, the whole training set was run through the transformations, providing slightly different segments from the base data. A summary of the label dataset can be found in Table 2.

#### Automatic classification of acoustic signals using a Convolutional Neural Network (CNN)

The approach to automatically classifying acoustic signals was based on a Convolutional Neural Network (CNN), a class of deep learning algorithms, that was trained to automatically perform image-based classification on the visual representation (spectrogram) of sounds^67^. This approach required input spectrograms with annotations identifying the sound classes to be classified. Network prediction consisted in the successive convolution of trained filters (or kernels) on the spectrogram image to extract relevant features for class prediction (classification). Over a series of epochs (iterations of the network over the training dataset), the CNN model described below was then trained using the binary cross-entropy loss function (with the loss summing over the labels and the batch) and the Adam optimizer^68^ to perform predictions on the presence of the classes within a spectrogram. The dataset used to produce the CNN classifier was split between a training and a testing dataset, each set containing data from the boat-based survey (different recording sessions), the research lodge (different days), and the várzea (equal split on a random selection) (see Table 2). Based on the output prediction, the classifier performance was evaluated.

#### Data pre-processing and CNN architecture

We used a convolutional neural network (CNN) with the architecture as shown in Supplementary Materials Figure S1. To compute the input features, the wave forms that were not already sampled at 96 kHz were first down-sampled to 96 kHz by low pass filtering and decimation by 2 or 4 depending on the original sampling frequency. Then all wave form data was combined and segmented into 5-s non-overlapping segments. The classifier operated in a frequency band from DC to 48 kHz. Each segment was Fourier transformed using a 2048 sample Hamming window with 1112 samples of overlap to compute the power spectral density (PSD). This resulted in a time–frequency matrix of 1024 by 1024 PSD values. The PSD values were log-transformed element wise. Then the frequency dimension of the matrix was Mel scale-transformed such that 1024 linear frequencies were mapped to 128 log-spaced Mel bands. Finally, the matrix was equalised over the time dimension (subtraction of the median for each Mel row) and then presented as input to the classifier. The first part of the CNN classifier consisted of 5 blocks that were identically structured, with each block containing two 2D convolution layers that were using the same number of 3 × 3 filters with the rectified linear activation function, followed by a max-pooling layer (using 2 × 2 filter without overlap). The number of filters was changed each iteration of a block: 32, 64, 96, 128, 160. All convolution layers were preceded by a batch normalisation layer. After the last convolution block, the resulting feature maps were reshaped to obtain a two-dimensional matrix which was run through two one-dimensional convolution layers with batch-normalization, each convolution with 256 filters, kernel size 1 and rectified linear activations. Finally, the per-class output was obtained through a 1-dimensional convolution layer with sigmoid activation.

#### Active learning procedure

**Figure 2:**
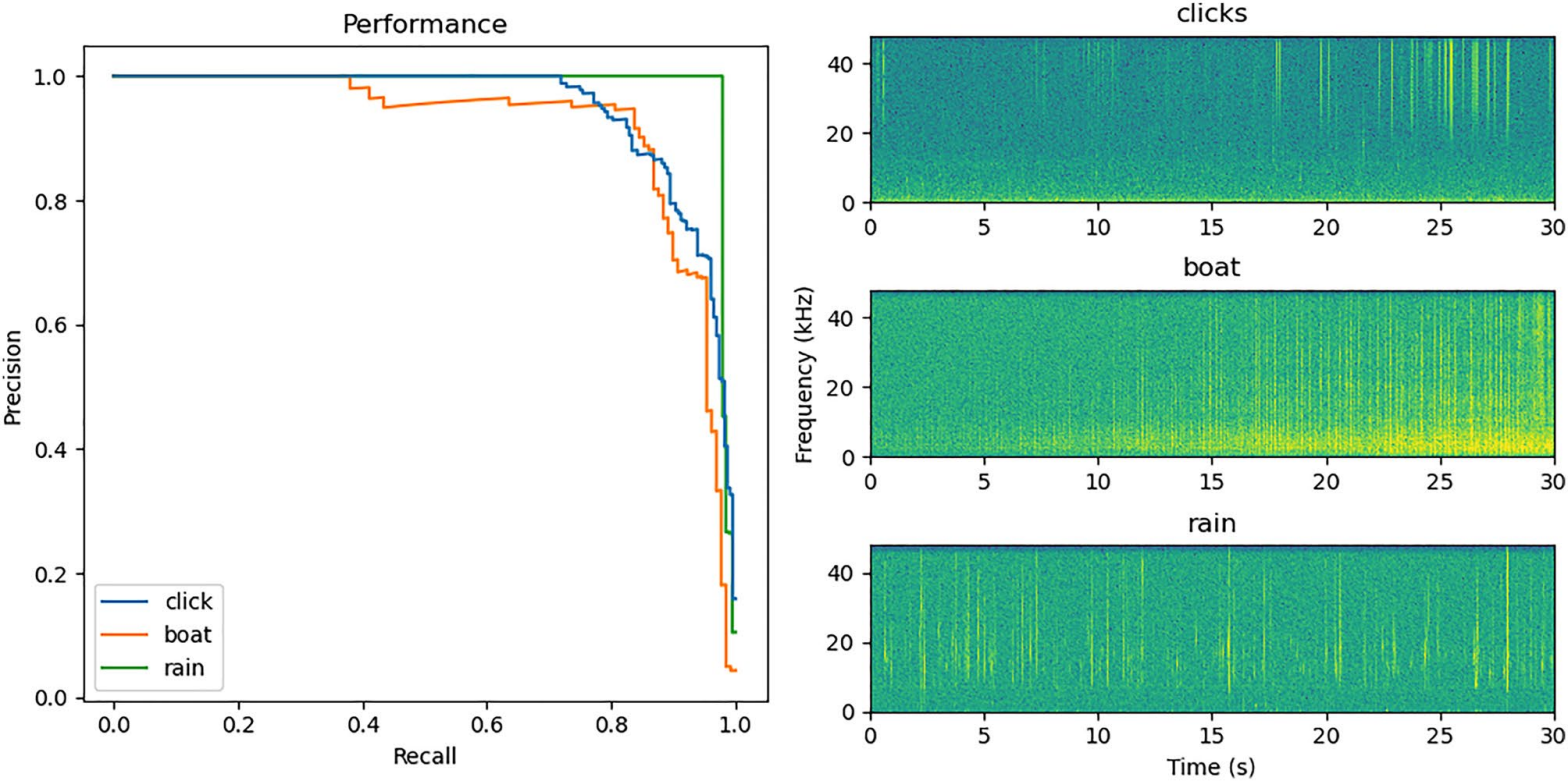
Precision-recall curves for class click, boat and rain (left) and spectrograms of the three corresponding impulsive sounds classified (right).

Considering the large dataset and relatively rare presence of some of the target signals, an active learning approach was followed. After first training using the boat-based and the Providence node dataset, the classifier model was evaluated on other data sets (Boca and várzea sites) to identify misclassifications, i.e. new sound types not present in the soundscape of the initial training data that conflicted with classified classes. Predictions from the initial model were manually checked for out-of-sample errors (generalisation errors). Custom Python scripts were created to automatically extract and display spectrograms of randomly selected positively classified sounds, above a threshold of the classification output selected from the model performance (see paragraph below). Misclassifications for the classes ‘*click’* and ‘*boat’* were identified through visual observation of spectrogram segments, and typically contained unrelated sounds with similar/overlapping acoustic characteristics to that of the sound types of interest (e.g. rain, see Fig. 2). The audio segments containing correctly classified and misclassified sounds were automatically extracted and combined with the initial training dataset for retraining of the model. An overview of sound labels by site used for initial classification and active learning can be found in Table 2.

#### Classification performance evaluation

The final classifier was trained with a data set consisting of the initial and active learning data sets, and classified three impulsive sound types: echolocation clicks, shipping noise and rain. First, we produced a summarised classification output calculated over a 5-s segment. The dimension of the (scaled) spectrogram submitted to the classifier was a 512 × 128 matrix (time x frequency bins). After the iteration of convolution-pooling layers, this was reduced to a 16 × 3 matrix, and classification was performed on each column producing 16 output values per class (3) per segment. After CNN training, the 16 values per class were summarised by taking the mean over the values between the 75^th^ and 100^th^ percentile as the segment-based classification result, referred to as Q75. After evaluating several options we found that the 75^th^ percentile had best performance for a signal that we expect to be repeated several times within the spectrogram. This approach should reduce the number of spurious high classification values under the assumption that our target classes have multiple high output values per 5-s segment.

Some performance metrics, for example accuracy, can be deceptive when considering the actual performance of a classifier, since the data are unbalanced (i.e., some classes are more prevalent than others in the dataset) and when models give the probability score^69^. Here, we preferred reporting on precision and recall, metrics that can provide a better insight when dealing with unbalanced class representation^69,70^. The precision (also called Positive Predicted Value, PPV) was defined as the fraction of observations predicted to be positive that were in fact positive. The recall (or True Positive Rate, TPR) was the fraction of observations classified as positive out of all positive observations (i.e. a probability of detection). A precision-recall curve was plotted wherein these two performance metrics were respectively plotted on the x and y axis for a sequence of decision thresholds. Then, the Average Precision (area under the precision-recall curve, AP) was computed for each class to evaluate the individual class performance. The global performance of the classifier over the three classes was computed using the mean average precision (mAP) through micro averaging, where all classification results from the different classes were combined to compute a single precision-recall curve, and then the AP from this curve was computed to give the mAP; this approach accounted for class unbalance.

$${\text{Precision}}\; \, \left( {{\text{PPV}}} \right) \, = {\text{ True}}\;{\text{ Positive }}/ \, \left( {{\text{True }}\;{\text{Positive }} + {\text{ False}}\;{\text{ Positive}}} \right)$$

$${\text{Recall }}\left( {{\text{TPR}}} \right) \, = {\text{ True}}\;{\text{ Positive }}/ \, \left( {{\text{True }}\;{\text{Positive }} + {\text{ False }}\;{\text{Negative}}} \right)$$

$${\text{True }}\;{\text{Negative}}\;{\text{ Rate }}\left( {{\text{TNR}}} \right) \, = {\text{ True}}\;{\text{ Negative }}/ \, \left( {{\text{True}}\;{\text{ Negative }} + {\text{ False}}\;{\text{ Positive}}} \right)$$

$${\text{Average }}\;{\text{Precision }}\left( {{\text{AP}}} \right) \, = \mathop \sum \limits_{t = 0}^{t = T - 1} \left[ {TPR\left( t \right) - TPR\left( {t + 1} \right)} \right] * PPV\left( t \right)$$

with T the number of thresholds and TPR(T) = 0, PPV(T) = 1.

### Amazon River dolphin presence and boat passage

#### Choice of optimal classification threshold

The entire dataset except the boat-based surveys (2931 h of recordings) was evaluated with the final model. For each 5 s segment, this gives 3 predicted scores for the 3 target classes (*click, boat, rain*). Validation outputs (Average Precision curves and scatter plots) of the final model were used to identify the optimal classification threshold for each sound type. The optimal threshold (or decision threshold) was determined as the value point of the Average Precision curve where both Precision and Recall are equal. Decision thresholds were then tested and evaluated per location on randomly selected classified segments from the entire dataset. The TPR and the True Negative Rate (TNR) were assessed and the thresholds were adjusted and re-evaluated if necessary. The decision threshold for each class and location was then compared to the prediction score of each segment classified, and the segment was assigned as positive for dolphin (*click* class), ship (*boat* class) or rain (*rain* class) presence if the prediction score was above the class threshold.

#### Metric of daily acoustic presence

Five second segments with CNN scores above the threshold were counted as acoustic occurrences of river dolphins. Daily acoustic presence is a proportion that was obtained as the daily duration with acoustic occurrences divided by the total daily recording duration. Reporting a proportion compensates for the differences in the recording duty cycles between the Providence node and the SM4 recordings. For the várzea sites, where dolphin passages were assumed to be infrequent and further away from the recording equipment with complex propagation conditions (flooded forest), the positive detections were manually checked.

#### Additional filters

For the várzea sites, dolphin detections typically had very low signal-to-noise ratios (SNR) compared to the river channel detections. This led to an increase in click misclassifications with the rain sound-type. As the final classifier has a higher performance for rain than clicks (see Results), the rain class was used as a posterior filter for misclassifications from all recording sites (e.g. any 5-s segment that contained a value above threshold both for click and rain sound-types was attributed to rain).

#### Co-occurrence of dolphins and boat

To investigate the spatio-temporal overlap of dolphins and boats, a co-occurrence count was calculated at Boca, the location with the highest acoustic occurrence of dolphins. This count corresponds to the number of minutes that contain both positive click classification and positive boat classification.

## Results

### CNN Classifier performance

The initial and the final classifier performance were assessed based on the precision recall curve for the 3 impulsive sound types: Echolocation clicks from river dolphins, engine noise from passing boats and rain.

The initial classifier had an Q75 Average Precision of 0.90. After the active learning procedure, tests with reduced segment length, and addition of the *rain* class, the final classifier had an Average Precision of 0.95. This corresponds to an overall increase of 5.5% on the Average Precision after the active learning procedure. Highest Average Precision was achieved from the *rain* class with 0.98. *Click* and *boat* classes also showed high performance with respective values of 0.95 and 0.92 (Fig. 2). Positive classification threshold for echolocation clicks was set to values shown in Table 3, selected from the semi-automated performance evaluation performed for each recording location. Table 3 also shows the corresponding TPR and TNR for a given threshold at a given location. *Click* TPR and TNR were between 0.88 and 1, while these values ranged from 0.94 to 1 for the *boat* class.

**Table 3:** Classification threshold selected for echolocation click and boat at each recording location, and associated TPR and TNR assessed on 60 randomly selected classified segments (see Methods).

Class	Recording location	Class threshold	Rain filter threshold	TPR	TNR
Click	Rato	0.85	0.90	0.96	0.91
	Aracazinho	0.85	0.99	1	0.88
	Juruazinho	0.85	0.99	1	0.88
	Boca	0.85	0.99	1	1
	Research lodge	0.85	0.99	0.96	0.94
Boat	Rato	0.98	NA	1	1
	Aracazinho	0.98	NA	1	1
	Juruazinho	0.98	NA	1	0.97
	Boca	0.97	NA	0.94	0.97
	Research lodge	0.98	NA	1	0.97

### Acoustic presence of river dolphins

**Figure 3:**
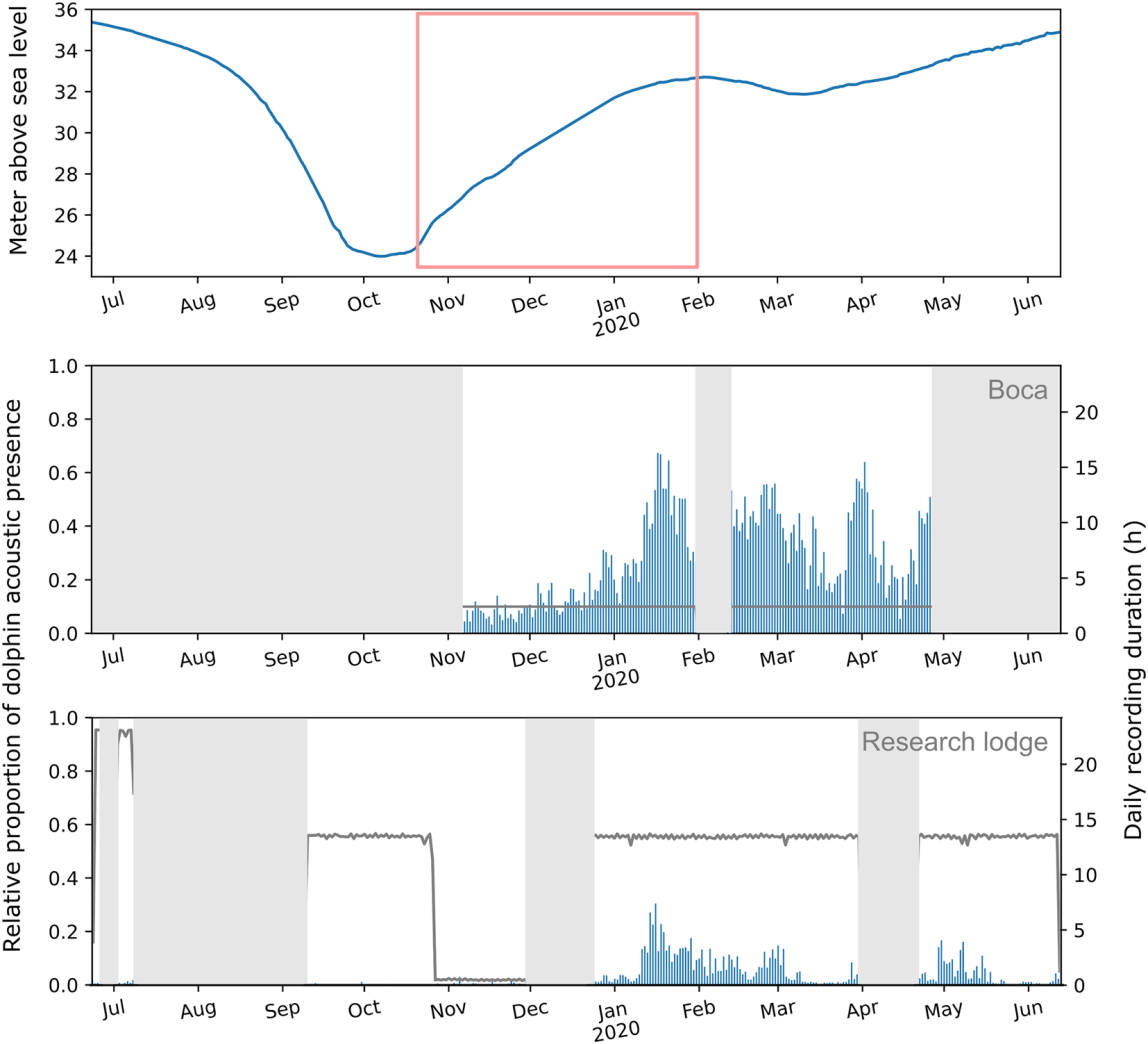
River dolphin acoustic presence at the ressaca and in the river channel (blue bars), based on CNN click classification. Water levels (top); Presence at the entrance of the river channel (middle); Presence at the Research lodge (bottom). The pink box indicates the rising water period. The grey line is the daily recording duration. Grey areas indicate an absence of recordings.

Acoustic detection of dolphins was frequent at the two sites located in permanent bodies of water (Fig. 3). At Boca (the *ressaca* location), dolphin presence based on echolocation clicks was detected over the full deployment period, from November 2019 to May 2020. Dolphin presence increased from November to the beginning of January 2020, when detections peaked briefly in mid-January (dolphin presence detected approximately 70% of the daily time). Detections peaked similarly at the end of February and at the beginnings of April and May (Fig. 3, middle). At the Research lodge (the *river channel* location), there was a very low level of detections in the beginning of July and from September to November Detections then started to increase until peak presence in Mid-January when dolphins were detected approximately 30% of the time. Between March and April and at the end of May, detections decreased to minimum values (Fig. 3, bottom).

The annual cycle of flooding between June 2019 and June 2020 (Fig. 3, top) showed the typical pattern of fluctuations between high and low water levels. From June to October, water levels decreased until reaching a minimum. This corresponded to a null or very low number of clicks detected at the Research lodge in the river channel. Later, at both sites (*ressaca* and *river channel*), dolphin presence increased over a 3-month period from November to January, with a clear synchronised peak in mid-January at the two locations. This time of the year corresponds to the rising water period of the annual cycle of flooding. In 2019, water levels rose 8 m from October to reach their highest level in mid-January (Fig. 3, top). Finally, during the following month (February to end of May), water levels remained high, and dolphins were detected regularly at both sites with similar variations in the rate of detection (matching peaks in March, April and May).

**Figure 4:**
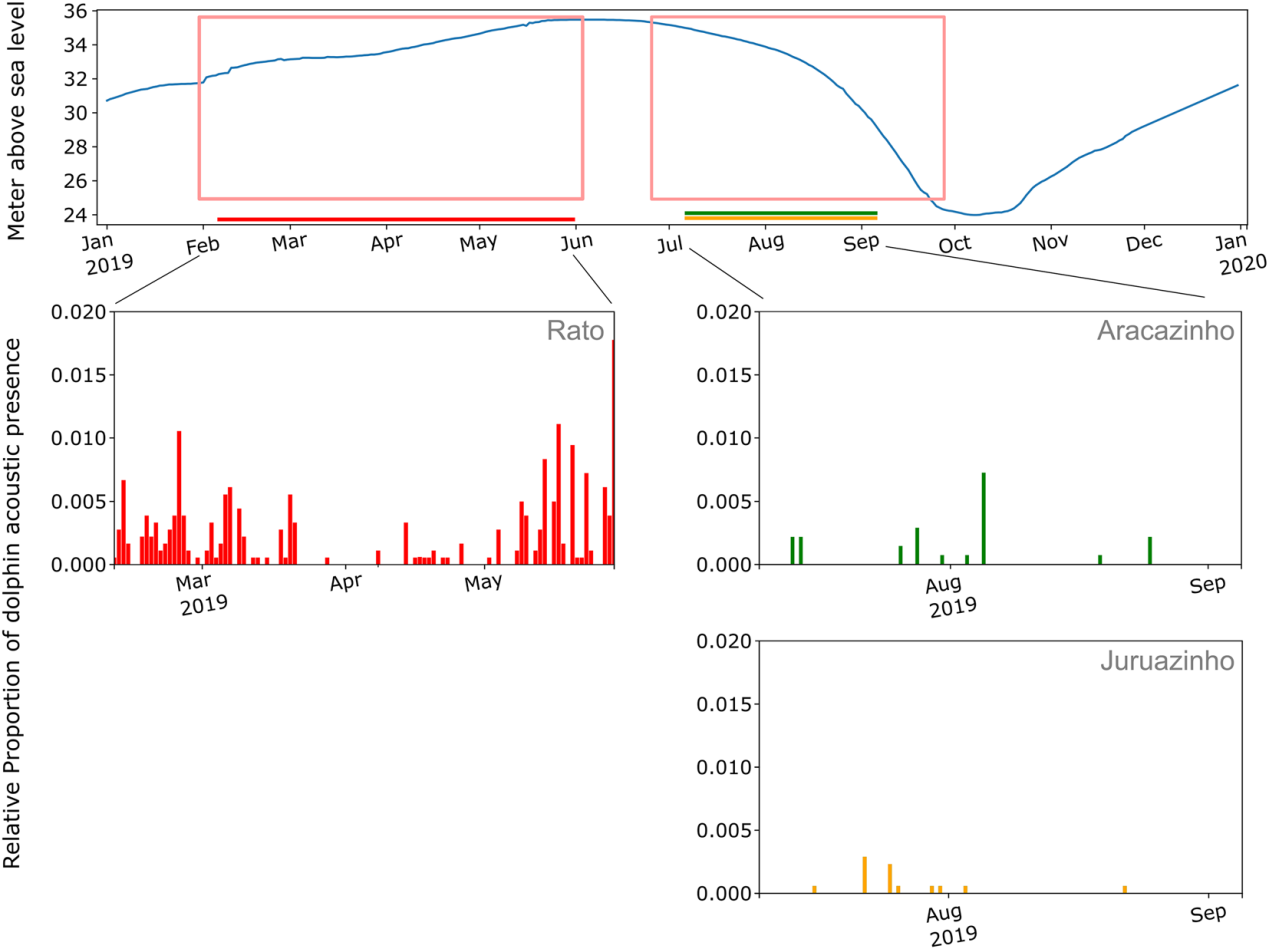
River dolphin acoustic presence at three periodically flooded sites, based on CNN click classification. Water levels (top); Dolphin presence at Rato (middle left); Presence at Aracazinho (middle right). Presence at Juruazinho (bottom right). The pink boxes indicate the high water period (left box) and the receding water period (right box).

River dolphin acoustic presence was lower in the periodically flooded sites than at the permanent bodies of water. At *Rato,* one the flooded forest site*,* dolphins were mainly present in February–March and in May, during 64% of the sampling days, with a maximum value of 1.7% of the daily time. The recording period corresponds to the end of the rising water period (Fig. 4, top). At *Juruazinho*, the other flooded forest site, during the 2 and half months of recording available, detection rates were very low and dolphin presence was detected essentially during 8 days (12% of the sampling days) between the end of July and the beginning of August. The detections were of the same order of magnitude at *Aracazinho*, the internal lake location (less than 0.6% of the time, 9 days of detections). In terms of water level, this period corresponds to the receding water, where water levels in early July began to drop to reach approximately 30 m above sea level in early September.

### Spatio-temporal co-occurence of river dolphins and boats

**Figure 5:**
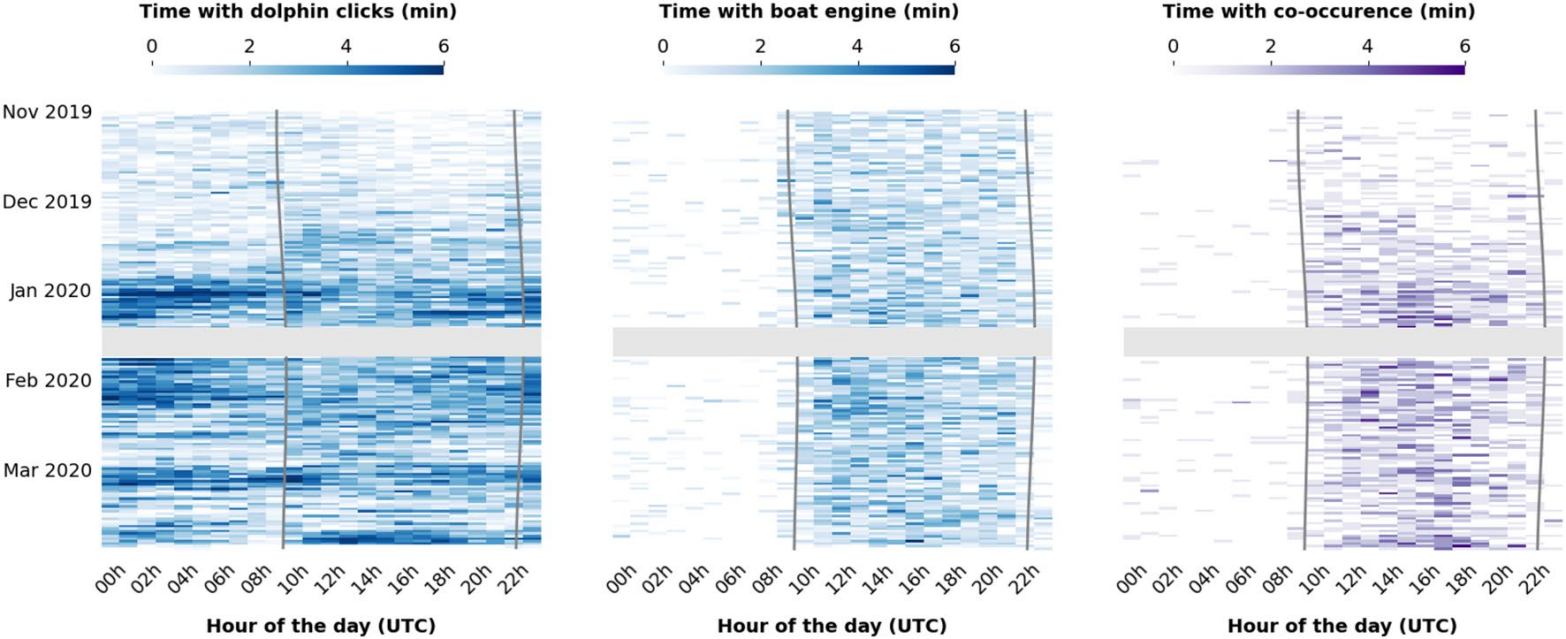
Acoustic presence of dolphin, boat, and co-occurence grouped by hour of the day at the Boca site. The dark grey lines represent dusk and dawn. The light grey areas represent a period without data collection (battery replacement). Please note the values of 6 min per hour is the maximum value that can be obtained based on the data collection duty cycle (1 min on, 9 min off, see the Methods section).

The dolphin activity varied strongly by month, but when they were present there did not seem to be a strong difference between day or night activity (Fig. 5, left). A Wilcoxon rank-sum test was used for each month to compare the distributions of the number of positively classified segments between day (dawn–dusk) and night (dusk–dawn), both scaled by the time period being measured in minutes as the duration of day and night changed over time (H0: the day/night activity is the same; H1: one time of the day has higher activity than the other). A non-parametric test was selected because the distribution shapes between day/night appeared to be different and not normal. Only February showed a significant difference between day/night (*p* = 0.00 with N = 18 as there was missing data at the start of the month). But for the other months such a difference was not found. Boat passages were almost exclusively detected during daylight hours without noticeable difference between the hours of the day or between months (Fig. 5, middle).

The temporal overlap between boats and dolphins was estimated through the co-occurence of segments with positive clicks and boat classification within a 1-min time period. Figure 5 shows relatively low values in terms of time with co-occurrence. Over the full 5-month dataset, the number of minutes with co-occurrence is low (mean 0.54, SD = 0.94), representing an average of 9% of the recorded time. These values remain low when computed over the 3-month period corresponding to the higher dolphin presence in January-March (mean = 0.67, SD = 1.05) with 11% of the recorded time.

## Discussion

The results of the CNN automatic classification of river dolphin echolocation clicks revealed patterns of presence in relation to the period of the annual water flood. In the bay and in the river channel, dolphin acoustic presence clearly increased during the period of rising waters, from November to January. This pattern was especially conspicuous in the bay (entrance of the river channel), where the daily acoustic presence rose from approximately 10% to 70% during this period. Interestingly, the main peak of acoustic presence was detected at both sites simultaneously. The synchronised detection peak at both locations suggests local population scale movements of dolphins entering the várzea from the main river through the river channel. These results are in agreement with published data on dolphin movements in the várzea in relation to the flooding cycle. Martin and da Silva^21^ reported the movements of the dolphins inside the RDSM through the river channel during rising waters based on visual surveys and radio-tracking data, with a rapidly increasing presence of dolphins peaking “at about a level of 10 m”*.* Our results show a similar pattern of detections, with a peak at rising water, when water levels reached 8 m above the lowest level. At high water levels (from February to June), dolphins remained present in the bay, where they were acoustically detected between 20 and 60% of the time. Dolphins were also regularly detected in the river channel, although not continuously, and with a considerably lower acoustic presence (0–15% of the time).

These findings further support the idea that bays formed at confluence areas are an important habitat for river dolphins. Mintzer et al.^5^ studied the seasonal movements of botos inside the RDSM estimating a transition probability between habitats, and characterised the entrance of the Mamirauá lake system as a core area for botos (i.e. where animals spend a maximum amount of time). Especially mother/calf pairs and immatures seemed to spend more time in the bays before moving back into the Mamirauá lake system at low/rising water. This preference was also demonstrated through a PAM study from the same location at the end of rising waters^43^. Tucuxis also seems to favour confluences^29,71^. The importance of this habitat appears to be shared by Amazonian River dolphin populations and subpopulations across their distribution^33,34^. A recent study covering several locations in both the Orinoco and the Amazon basin highlighted that the highest dolphin densities for both Amazonian River dolphin species were found in the confluence areas, with densities averaging 23 and 16 ind./km^2^ for botos and tucuxis respectively, and reaching 61 and 64 ind./km^2^ in the confluences of the Mamirauá Reserve^72^.

Additionally, river channels appeared to be used by dolphins, especially botos, as a gateway to access remote parts of the várzea. Results from past PAM study in the RDSM investigating dolphin click trains and trajectories showed that the animals mainly used the Mamirauá channel as a passage to other locations of the várzea^43^. From this channel, botos could access either permanent lakes connected (e.g. Mamirauá lake) or disconnected from the riverine system at low water (e.g. Rato lake) and seasonally flooded lakes (e.g. Juruazinho and Aracazinho lakes). Tucuxis are also known to be present in the channel, although limiting their use of the channel to the lower part, closer to the main river^29^.

Thus, the difference between detection values at the two sites (situated 10 km apart on the same river channel) could be explained not only by a difference in the number of dolphins present but also by a difference in their habitat use. The entrance of the channel is a bay (*ressaca*), close to a confluence of two major waterways, the Solimões (upper part of the Amazon) and the Japurá rivers. The local environmental conditions create favourable low-current prey-rich habitats for the dolphins. Higher acoustic activity could reflect either an increase in time that individual dolphins spent in the area, an increase in the number of dolphins using this area, but also an increase in the acoustic activity due to the higher click production used for foraging compared to travelling behaviours^47,73,74^.

Dolphin detections at high water in the flooded forest (Rato) were very low in terms of duration (less than 2% of the daily time) but regular in terms of presence (i.e. number of days). The Rato flooded forest site is an access to the Rato lake and dolphin detections likely reflect the regular passage of botos to the remote parts of the floodplains. Várzea lakes, especially the ones with floating vegetation that provide refuge for a great variety and abundance of fish, are also a favourite habitat of river dolphins^21^. Nevertheless, due to the difficulties to penetrate the intricate flooded forest ecosystem with boats, there is very little information on the distribution of dolphins in the mosaic of floodplain habitats. Even using alternative monitoring techniques, such as tracking animals through tags, data on dolphin distribution once they leave the river channel is excessively difficult to collect. In a study using VHF transmitters on 24 botos, Martin and da Silva^21^ reported that during high waters the tagged botos were out of range up to 100% of the time, preventing their localization inside the area. Our results indicate here the regular use of flooded forest passages connecting várzea channels to internal lakes by the dolphins during high waters.

Females botos with calves and immatures animals spend more time in the várzea habitats than males^21^. One of the reasons is that várzea habitats provide access to rich prey resources. Floodplain systems, which combine high levels of habitat complexity with nutrient-rich waters, host a great diversity of fish associated with high biomass^17,75,76^. Another hypothesis is that várzea habitats seem to grant shelter against males’ aggressive behaviour, especially towards calves^77,78^. Finally, habitats such as internal lakes, flooded forests and small channels provide resting areas with lower currents^5,21^ that are usually favoured by river dolphins. This unique and beneficial combination of environmental conditions make várzea habitats of major importance for females with calves and immatures, and therefore for boto populations survival.

From July to September, at the end of high water and at falling waters, our study collected data on two sites: one inside the flooded forest (Juruazinho), and one in an internal lake (Aracazinho). The detection levels were very low and of the same order of magnitude at both sites, with 12% of the days with dolphin detections, mostly occurring in July and early August. Unfortunately, no data could be collected during the same time period at the other study sites, and it was not possible to draw strong conclusions about the relationship between the limited presence of dolphins and the decrease of water levels. Nevertheless, our results are aligned with known dolphin movements outside the várzea. Both river dolphin species are known to move back into the main rivers during this time period, following fish movements outside the floodplains, and anticipating the upcoming risk of entrapment^5,21,29,79^.

Quantifying the extent of spatiotemporal co-occurrence between dolphins and boats in freshwater environments is of critical importance, especially in core areas where the animals spend a great amount of their time budget in vital activities such as foraging. Passage of boats in these spatially restricted areas introduces engine cavitation noise in the underwater environment. Chronic stress, masking effects, behavioural and acoustic responses were reported for marine populations in the open ocean^80^ and it can be assumed that such responses are even stronger in freshwater systems of (e.g. rivers, channels, bays) that are spatially restricted and provide less opportunities for animals to evade disturbance. Our results show that at Boca, where continuous and significant dolphin presence was detected during the 5-month recording period, the level of co-occurrence was approximately 10% of the recorded time. Nevertheless, the data was collected on a 10% duty cycle, and can only provide an estimation of how much boat traffic overlaps with dolphin presence. It is likely though that at this location the level of disturbance is reduced due to the low level of boat traffic (essentially fishermen from local communities and tourism boats that pass by a few times per week). However, effects of underwater noise on river dolphin populations are remarkably understudied^80^ and so far only a handful of publications have addressed the effects of shipping traffic on freshwater cetacean populations^81–83^. Therefore, the effects of noise exposure representing 10% of the time spent by river dolphins in core areas can not be evaluated. Continuous recordings are needed to accurately assess the overlap in boat and dolphin presence and evaluate the potential disturbance caused by this source of underwater noise. Further studies will be conducted at other confluence habitats regularly used by the river dolphins, where boat traffic is higher (e.g. Lake Tefé).

The CNN method developed here classified three sound types (echolocation clicks from river dolphins, boat noise from engine cavitation, and rain), with a mean Average Precision of 0.95. The sounds are represented as spectrogram images and this demonstrates the validity of using an image-based approach for classifying and discriminating underwater acoustic events of impulsive nature. Once labels were created (a time-intensive task), our CNN-based workflow required only a few pre-processing steps. Furthermore, with the integration of on-the-fly data augmentation, this workflow allowed to train initial models for undersampled classes. These models were used to automatically retrieve additional true positive (sound type of interest) and false positive (misclassifications) examples in an active learning loop in order to swiftly strengthen the model performance.

There is an increasing interest in using convolutional neural networks to automatically detect and classify odontocete echolocation clicks. Some studies focus on classifying single clicks in a single class classifier and achieve high performance^60,84^. However, compared to monospecific recordings (i.e. containing one sound class), classification tasks using soundscape recordings (i.e. containing multiple sound classes) represent an important challenge for classification algorithms. Recent classification contests demonstrated that the performance scores achieved on soundscape recordings were 4 times smaller than on monospecific recordings^85^. We chose to base our workflow on 5-s soundscape recordings with the objective of furthermore developing a general classification model for different types of sounds (impulsive or tonal) from biological (Amazon aquatic species), anthropogenic (e.g. boat) and natural (e.g. rain) sources.

## Conclusion

This study demonstrates the suitability of using CNN-based classification to automatically detect river dolphin echolocation clicks in the complex soundscape of freshwater habitats. The efficiency and speed of the CNN method allow to analyse the totality of the data collected without having to subsample as usually done for manual analysis, making it possible to detect major movements of dolphins in the study area, and rare passages in specific habitats or seasons. The use of Passive Acoustic Monitoring coupled with automatic analytical methods such as CNN-based classification of dolphin signals can efficiently increase our knowledge on endangered dolphin populations across a range of flooded habitats, especially in remote and understudied habitats of flooded forest and lakes, and allows to precisely time the movements of river dolphins between várzea habitats in relation to the flooding pulse. The classifier in this study was extended to include automatic detection of boat passages in dolphin core areas to assess the extent of underwater noise disturbance on river dolphins.

Our study calls for a generalisation of the use of PAM inside the mosaic of floodplain habitats to understand habitat preferences and requirements of river dolphins, especially the boto females and calves. Practical applications in forecasting the dolphins’ response to habitat loss and degradation (e.g. deforestation for pastures, plantations, selective logging, …) will contribute to the management strategies of the aquatic-terrestrial transition zone (ATTZ), critical for the maintenance of habitat connectivity^16,86^. Another area of applications is towards developing and implementing standardised protocols to monitor distribution shifts in relation to the recent amplification of drought and flood events in the Amazon basin^87^. As sentinel species of the aquatic systems they inhabit, river dolphins can constitute an early detection system of ecosystem unbalance^26^.

## Supplementary Information

Supplementary Figure S1.

## Data Availability

The datasets generated during the current study are available from the corresponding author on reasonable request.
