# Supplementary material for: Towards automated long-term acoustic monitoring of endangered river dolphins: a case study in the Brazilian Amazon floodplains
Source: Sci Rep. 2023 Jul 27;13:10801. doi: 10.1038/s41598-023-36518-1 (PMC10374533; doi:10.1038/s41598-023-36518-1)

SUPPLEMENTARY INFORMATION

Towards automated long-term acoustic monitoring of endangered river dolphins: a case study in the Brazilian Amazon floodplains

Florence Erbs[1], Marina Gaona[1,2], Mike van der Schaar[1], Serge Zaugg[1], Emiliano Ramalho[2], Dorian Houser[3], Michel André[1*]

1 Laboratory of Applied Bioacoustics. Spain.
2 Instituto de Desenvolvimento Sustentável Mamirauá. Brazil.
3 National Marine Mammal Foundation. United States.
Correspondence : michel.andre@upc.edu

Figure S1. Representation of the Convolutional Neural Network architecture

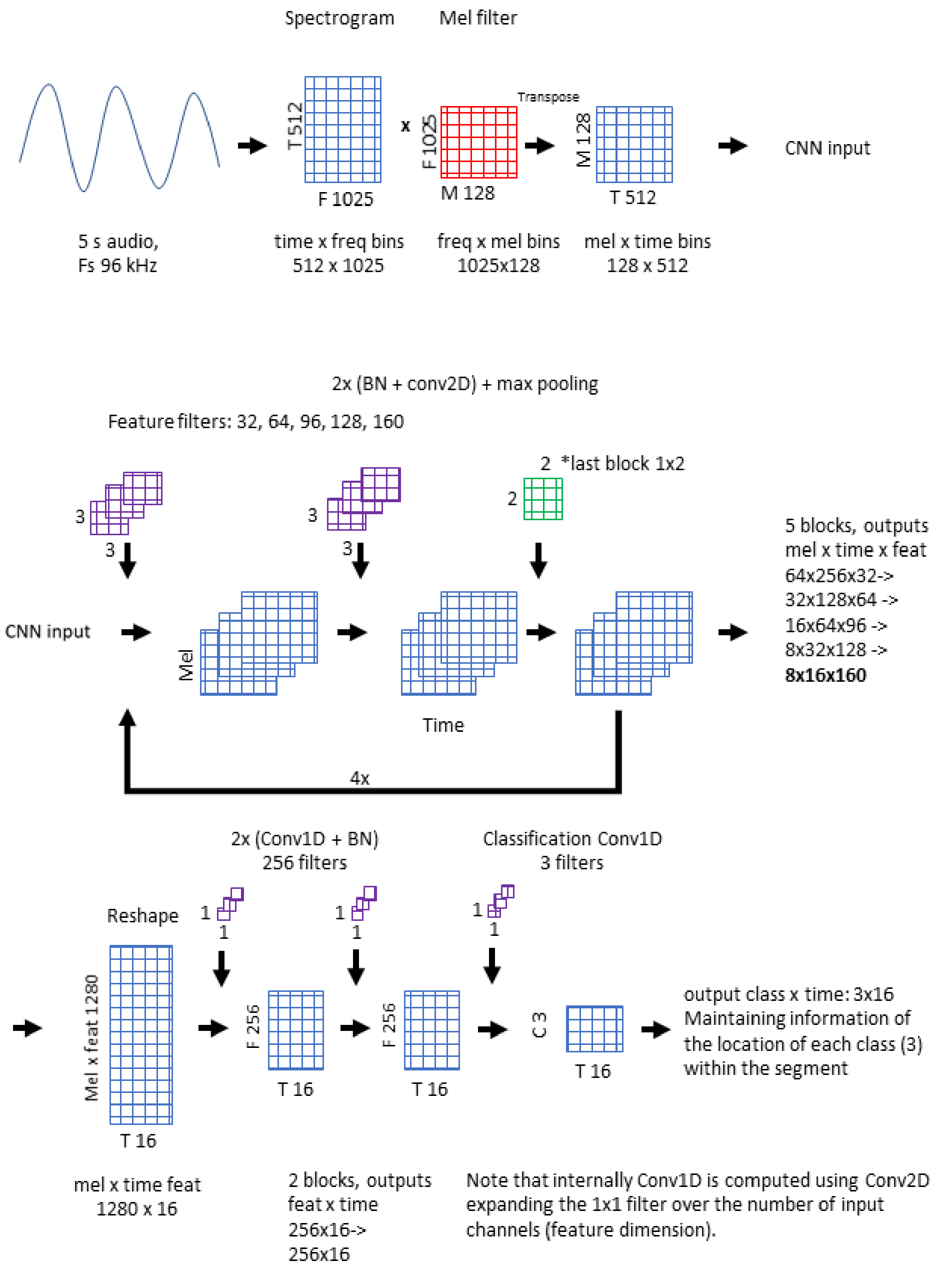

Supplement: Supplementary file 1 — Supplementary Figure S1. [file 41598_2023_36518_MOESM1_ESM.pdf]
